# Metabolic interactions and growth dynamics of *Bifidobacterium* strains in response to human milk oligosaccharides and lactose

**DOI:** 10.3389/fmicb.2026.1805620

**Published:** 2026-09-01

**Authors:** Stefan Ratering, Sylvia Schnell, Christian Borsch, Sinéad T. Morrin, David R. Hill, Rachael H. Buck, Silvia Rudloff

**Affiliations:** 1 Department of Applied Microbiology, Justus Liebig University Giessen, Giessen, Germany; 2 Department of Nutritional Science, Justus Liebig University Giessen, Giessen, Germany; 3 Abbott, Nutrition Division, Columbus, OH, United States; 4 Retired, Columbus, OH, United States; 5 Department of Pediatrics, Justus Liebig University Giessen, Giessen, Germany

**Keywords:** 2’-fucosyllactose, 6’-sialyllactose, bacterial growth, co-cultures, fermentation, lactate, short chain fatty acids

## Abstract

Bifidobacteria are major representatives of the intestinal microbiota in infants typically identified as characteristic of healthy breastfed infants. Their establishment is driven by the presence of carbohydrates, namely human milk oligosaccharides (HMOs). Metabolic products of HMO utilization by the infant microbiota, such as short chain fatty acids and lactate, are associated with health benefits for the host. In the present study, we investigated the growth and acidic metabolites produced by infant typical *Bifidobacterium* strains *B. longum* subsp. *infantis*, *B. breve* and *B. bifidum* as single cultures as well as in co-cultures. The HMOs, 2’-fucosyllactose (2’-FL) and 6’-sialyllactose (6’-SL) were used as carbohydrate substrates given separately and together or as a mix with lactose and were compared with lactose alone and glucose as standard carbohydrate supplements in media. Bacterial growth of single cultures and co-cultures showed differences in growth curve and optical density at 36 h depending on carbohydrates. The best growth was reached in media supplemented with equimolar amounts of 2’-FL and lactose and accompanied by high concentrations of organic acids in all single and co-cultured *Bifidobacterium* strains as determined by HPLC analysis. When 6’-SL was supplemented, *B. bifidum* was the only strain able to grow in single cultures. In co-cultures, however, its presence affected the growth curves of mixed cultures although its own proportion of cells after 36 h remained as low to that of an estimated 10% of all bifidobacterial cells as determined by qPCR. The production of bacterial metabolites was more complex with 6’-SL and in combination with 2’-FL than with any other carbohydrate substrate. These results indicate that bacterial growth on specific HMO substrates in single strain cultures is not predictive of the potential production of organic acids in mixed strain cultures. The interaction of multiple *Bifidobacterium* strains and their cross-feeding properties when exposed to specific substrate compositions may affect the production of key metabolites important for infants and in later life.

## 1 Introduction

An intestinal microbiota dominated by *Bifidobacterium* species is associated with numerous health benefits (Lawson et al., 2020). The colonization of the infant gut by bifidobacteria is linked to their vertical transfer from mother to infant via the vaginal tract and breast milk (Collado et al., 2016). Bifidobacteria are prominent in the infant’s gut representing about 60–70% of all gut bacteria in healthy breastfed infants. The introduction of solid foods during weaning is associated with a significant reduction in bifidobacterial populations. Nevertheless, bifidobacteria continue to dominate the infant fecal microbiota during the first year of life, despite a progressive decline in their abundance over time. In adulthood, they continue to represent a significant proportion of the intestinal microbiota of healthy subjects, however in much smaller numbers and limited to certain species, such as *B. bifidum* and *B. breve* (Yatsunenko et al., 2012; Arboleya et al., 2016). Recent reports indicate that the prevalence of bifidobacteria within the infant microbiota significantly varies among geographical regions with *Bifidobacterium* species lacking in about 25% of infants in the US. This observation may have several explanations, including a higher prevalence of non-vaginal birth by cesarean section, a low breastfeeding rate or a lower prevalence of *Bifidobacterium* species due to a wide use of antibiotics (Taft et al., 2022; Laursen and Roager, 2023; Jarman et al., 2025). In general, bifidobacteria are able to differentially utilize a number of carbohydrates as carbon sources which may explain the occurrence of specific bifidobacterial species especially *B. longum* subsp. *infantis*, *B. breve*, and *B. bifidum* in the gut of infants in particular those fed breast milk (Xiao et al., 2024). Human milk not only contains high amounts of lactose (65–70 g/L) but also a large variety of oligosaccharides at concentrations of about 5–20 g/L (Bode, 2012) which may serve as substrates for *Bifidobacterium* species owing to their inability to be digested by human enzymes and thus reach the lower parts of the intestine as substrates for the gut microbiota (Garrido et al., 2016; Katayama, 2016; Yamada et al., 2017; Milani et al., 2017).

These human milk oligosaccharides (HMOs), of which about 200 different compounds have been identified in breast milk, are composed of the monosaccharides glucose, galactose and fucose as well as the amino sugars N-acetyl-glucosamine, N-acetyl-galactosamine and N-acetyl-neuraminic acid (sialic acid). All HMO structures are built up from a lactose moiety at the reducing end which can be elongated by the addition of lactosamine or lacto-N-biose. Lactose or the elongated oligosaccharides can further be fucosylated and/or sialylated in specific linkages. Despite their complexity, small HMOs such as 2’-fucosyllactose (2’-FL) or 6’-sialyllactose (6’-’SL) are quantitatively of major importance with great individual variability in concentrations from 0.7 to 4.8 g/L (corresponding to 1.5–10 mM) for 2’-FL depending on the presence of the fucosyltransferase 2 gene (FUT2) and 0.3–1.0 g/L for 6’-SL (Bode, 2012; Thurl et al., 2017; Triantis et al., 2018). However, it has been shown that milk from about 20% of mothers lacking 2’-FL due to an inactive fucosyltransferase 2 gene (FUT2) was associated with decreased concentrations of fucosylated HMOs (Kunz et al., 2017) and a delayed colonization of the infant’s gut with bifidobacteria (Lewis et al., 2015).

*Bifidobacterium* species, such as *B. longum* subsp. *infantis*, *B. breve*, and *B. bifidum* showed various abilities to metabolize HMOs, and these have been shown to be structure specific (Asakuma et al., 2011). For example, *B. longum* subsp. *infantis* possess multiple genes associated with ABC transporters and enzymes to metabolize them intracellularly. *B. breve* generally consumes only certain HMO types or their cleavage products by intracellular mechanisms. *B. bifidum* can also grow on all types of carbohydrates, mainly driven by extracellular surface-anchored glycosyl hydrolases to cleave glycosidic bonds in HMOs and internalize few molecules such as lactose and lacto-N-biose (Pröschle-Donoso et al., 2025). These different utilization strategies could have implications on the colonization of the gut, as extracellular mechanisms could favor cross-feeding, e.g., for sialic acid or fucose as cleavage products from HMOs such as 2’-FL and 6’-SL. This is important to note since infant formulas are regularly supplemented with various oligosaccharides that are structurally identical to HMOs, mainly 2’-FL, 3-FL, 6’-SL, 3’-SL, lacto-N-tetraose and/or lacto-N-*neo*-tetraose) either as single oligosaccharides or as mixtures of 2 or more (Amin et al., 2025).

In studies investigating the impact of HMO supplementation on gut microbiota, bifidobacteria are often analyzed using fecal samples or *in vitro* using one or two *Bifidobacterium* species. Often, bifidobacteria are only identified on the level of the genus but not differentiated between the various species occurring in the infant intestinal microbiota, thus neglecting their different potential to produce metabolites such as various short chain fatty acids (SCFA) and their interactions in more complex bacterial communities.

Previous studies have shown that the co-culture of *Bifidobacterium* (e.g., *B. infantis*, *B. breve* and others) using HMOs (e.g., 2’-FL and 6’-SL) as substrates revealed fermentation products differing from those that were produced from single strains (Gotoh et al., 2018; Tsukuda et al., 2021). In this study, cross-feeding was observed between different strains due to exo- and endoglycosidases differentially expressed in various strains. An increased metabolic activity in a co-culture of *B. longum* subsp. *infantis* with *B. breve* was indicated by a lower pH in the steady-state phase. This was postulated to occur due to an increased production of SCFA, e.g., after 2’-FL metabolism which may be even further enhanced by *B. bifidum* possessing extracellular fucosidases and sialidases (Nishiyama et al., 2018). In mixed cultures of *Bifidobacterium*, this may lead to outgrowth of other strains that are able to take advantage of the ability to use substrates resulting from exoglycosidic activity from other strains (Katoh et al., 2020). This broad set of glycohydrolases of *B. bifidum* may in part explain its presence through a wide range of age groups; however, it does not dominate the infant’s gut (Centanni et al., 2019; Sakanaka et al., 2019; Katoh et al., 2020).

Thus, the presence, but not necessarily the cell number of different *Bifidobacterium* species may be associated with their capacities to produce metabolic products like SCFA and lactate as organic acids which are associated with multiple positive health outcomes. Therefore, the efficiency of the bacterial use of HMOs and other carbohydrates may serve as sufficient markers to be used to observe the complex interactions of bacterial species and their production of beneficial acidic fermentation products when grown in co-cultures with mixtures of substrates. These acidic metabolites, i.e., lactate and other organic acids including the SCFA acetate, propionate or butyrate have distinct health effects apart from their ability to decrease the pH in the lower intestine, creating a less favorable environment for potential pathogenic bacteria. Specifically, lactate deriving from the exoglycosidic activity of microbes such as *Bifidobacterium* species, via metabolizing HMOs or lactose present in breast milk, could be further used as an energy substrate, or/and as a signaling molecule to the brain thus affecting neurodevelopment in early life (Cai et al., 2022). The SCFA acetate and propionate are major microbial metabolites and serve as substrates and regulators of infant energy metabolism and as signaling molecules driving cell differentiation and connections between the microbiome and the adaptive immune system. *Bifidobacterium* species and their metabolic products may thus affect infection outcomes in early life or the development of atopic disease (Kunz et al., 2000; Rudloff and Kunz, 2012; Bode and Jantscher-Krenn, 2012; Kim et al., 2014; Triantis et al., 2018; Bui et al., 2025).

Although the metabolic pathways of single *Bifidobacterium* strains for HMOs are well characterized, the interactions between multiple strains under conditions with more than one carbohydrate substrate, remain poorly understood (Sela and Mills, 2010; He et al., 2025). Here, we aimed at investigating different bifidobacterial species as single strains and in co-cultures to determine the relationship between the metabolic output of organic acids and various carbohydrate substrates and combinations thereof. The main hypothesis of the present study was that the interaction between the major *Bifidobacterium* strains comprising the human milk fed infant’s gut microbiota leads to different growth behavior as well as different output of metabolic acids such as SCFA and lactate when competing for different carbohydrate substrates. We further hypothesized that bacterial growth and a) the proportion of specific strains, b) the pattern and the amount of organic acids as bacterial metabolites produced, as well as c) the impact of substrate mixtures can be used as a predictor for the efficiency of nutritional interventions such as the supplementation of pro- and prebiotics.

## 2 Materials and methods

### 2.1 Bacterial strains and culture media

Three *Bifidobacterium* species were selected for the study. The strains *B. bifidum* JCM 7004, *B. bifidum* JCM 1254 and *B. breve* JCM 7019 were obtained from the Japanese strain collection (JCM Japan Collection of Microorganisms, Ibaraki, Japan), the strain *B. longum* subsp. *infantis* DSM 20088 from the German strain collection (DSMZ-German Collection of Microorganisms and Cell Cultures GmbH, Braunschweig, Germany). DSMZ medium 58 (*Bifidobacterium* medium) was used to cultivate the strains. The composition of the medium was as follows: in 950 mL deionized water, 10 g casein peptone (tryptic digest), 5 g yeast extract, 5 g meat extract, 5 g tryptic soy broth, 2 g K_2_HPO_4_, 0.2 g MgSO_4_ × 7 H_2_O, 0.05 g MnSO_4_ × H_2_O, 1 mL Tween 80, 5 g NaCl, 0.5 g Cysteine-HCl × H_2_O, 40 mL salt solution and 4 mL resazurin (25 mg in 100 mL). The salt solution contained 0.25 g CaCl_2_ × 2 H_2_O, 0.5 g MgSO4 × 7 H_2_O, 1 g K_2_HPO_4_, 1 g KH_2_PO_4_, NaHCO_3_ 10 g and 2 g NaCl in 1,000 mL deionized water. Autoclaving, cooling under N_2_/CO_2_ (10%/90%) atmosphere and anoxic filling of the medium into glass tubes or screw cap bottles was carried out as described in Widdel and Bak (1992). After cooling and before filling, the cysteine solution was added to the medium under sterile conditions, and the pH was adjusted with sterile NaOH (8 M) to a pH of 6.8. The bottles were filled to the edge with medium and sealed with screw caps with a Teflon seal. The tubes were filled with 5 mL medium and rinsed directly with N_2_/CO_2_ (10%/90%) using the Hungate needle and then sealed airtight with butyl stoppers. Strain maintenance was carried out in the 50-mL screw cap bottles and the growth experiments in the 20-mL glass tubes with 5 mL medium. For strain maintenance, a sterile glucose solution (1 M) was added to the screw cap bottles to achieve a final concentration of 5 mM.

### 2.2 Quantification with real-time PCR

Quantitative real-time PCR was used to determine the mixing ratio of the species at the beginning of the growth experiments and for quantification of the mixing ratio after the 36 h. The DNA was extracted from the bacterial pellets obtained after centrifugation of an aliquot of culture medium. First, a standard for the quantification using real-time PCR was prepared from each *Bifidobacterium* species. For this purpose, the cultures were allowed to grow in screw cap bottles with 5 mM lactose at 37 °C and after 36 h the culture was harvested by centrifugation, and the DNA was extracted from the pellet and purified according to the protocol of Quiroga et al. (2024). The DNA was used to amplify selective gene sequences with primers specific for the respective species. The primers developed by Kim et al. (2020) were as follows: *B. longum* subsp. *infantis* (ABC transporter permease; Infantis-F ATG ATG CGC TGC CAC TGT TA, Infantis-R CGG TGA GCG TCA ATG TAT CT), *B. bifidum* (Conserved hypothetical protein containing Ig-like domain; Bifidum-F CTG GCA GCC GTG ACA CTA CT, Bifidum-R TGA ACT GGC CGT TAC GGT CT), *B. breve* (Serine hydrolase; Breve-F TCA TCA CGG CAA GGT CAA GA 111 Breve-R GGC CAG AAC AGC TGG AAC AA). The amplicons were purified, and the correct length and specificity were checked using agarose gel electrophoresis. The amplicons were then cloned as described in Kampmann et al. (2012a) to ensure that the correct section was amplified, the cloned fragments were sequenced (LGC, Berlin, Germany). The copy number of the partial genes of the standard was calculated as described in Kampmann et al. (2012b). Since these genes occur in a single copy in the genome of the bifidobacteria, the copy number corresponds to the cell number.

To precisely mix the cultures for the growth experiments with more than one strain, it was necessary to determine bacterial cell number promptly. Therefore, the copy numbers of the individual *Bifidobacterium* species determined by real-time PCR were used for the standard curve against optical density (OD). For the standard curve, the 3 cultures of the individual *Bifidobacterium* species were grown in 50-mL screw cap bottles containing 5 mM lactose for 36 h at 37 °C in triplicate. A dilution series of the culture was prepared with sterile medium using 5 mL culture, 4.0 mL culture + 1 mL medium, 3.5 mL culture plus 1.5 mL medium, 3 mL culture plus 2 mL medium and 2 mL culture plus 3 mL medium. During the preparation of the dilution, the cultures or dilutions were kept on ice to prevent further growth. After thorough mixing, the OD of each dilution was measured using a spectrophotometer (Genesys 10S UV-VIS, Waltham, MA, United States) and the exact volume removed was noted. The remaining volumes of the dilutions were centrifuged at 13,780 × g and 4 °C, and the pellets were frozen until DNA extraction. DNA was isolated and purified from the pellet as described above. A separate quantitative real-time PCR was performed for each *Bifidobacterium* species. The DNA for the standard curves of each species was serially diluted 10-fold in the range from 10^2^ to 10^7^. For quantitative real-time PCR, 5 μL SYBR Green JumpStart Ready mix (Sigma-Aldrich, St. Louis, United States) plus 0.4 μL of the respective forward (10 μM) and reverse primer (10 μM), 2.2 μL PCR water and 2 μL of the DNA were mixed in a final volume of 10 μL. The DNA (standard and samples) was measured in quadruplicate using a Rotor Gene Q (Qiagen, Hilden, Germany) with the following program: 15 min 95 °C and 35 cycles of 30 s at 94 °C, 30 s at 60 °C, 60 s at 72 °C and 20 s at 84 °C. The software Q-Rex v1.1.04 (Qiagen, Hilden, Germany) was used for normalization, quantification and calculation of the copy number. To calculate the factor for converting the optical density into the copy number of the specific genes, the copy number was plotted against the OD, and the linear equation was determined (Origin Lab Cooperation, Northampton, United States) (Supplementary material S1).

### 2.3 Growth experiments

The experiments were carried out with various substrates and species and combinations of these substrates and species as shown in Table 1. The major milk oligosaccharides, 2’-FL [Fuc(α1–2)Gal(ß1–4)Glc] and 6’SL [NeuAc(α2–6)Gal(ß2-4)Glc] were obtained from Biozol Diagnostica (Hamburg, Germany), and lactose [Gal(ß1-4)Glc] (Riedel-de-Haen, Seelze, Germany) and glucose were used as controls, as well as a preparation without additional carbohydrate substrate, only with the substrates such as casein and yeast present in the medium. These substrates were supplemented to the media in a mid-physiological concentration of 5 mM for 2’-FL and isomolar for 6’-SL, when used as single substrates and in combinations as 2’-FL/6’-SL at a total of 5 and 10 mM, and as 2’-FL/lactose mixture at 10 mM in total. For the bacteria, all strains were tested individually with the substrates, then two strains combined and all three species together (Table 1). For the combination with several species and *B. bifidum*, strain JCM 7004 derived from infant gut was selected. A preculture with 5 mM glucose was grown at 37 °C for 36 h to inoculate the growth experiments. In the experiments with single cultures, the culture tubes were inoculated directly from the preculture. In the experiments with mixed cultures, the OD of the precultures was measured with a spectrophotometer prior to the growth experiment. The cell number per mL of culture medium was calculated using the factor of the standard curve (OD against cell number) for the respective species (Supplementary material S1). According to these calculations, mixed cultures were adjusted to have the same initial cell number. This mixed preculture was transferred into a sterile 20-mL glass tube and, after gassing with N_2_/CO_2_ (10%/90%) and sealing with butyl plugs, growth experiments were carried out in 5 mL medium. At the beginning of the experiment, the substrate was added to the glass tubes with anoxic medium in appropriate concentration and the calculated amount of preculture from the dilution using a sterile glass pipette. The medium was carefully mixed, 0.2 mL were removed, and the OD of the culture was determined at 500 nm using a spectrophotometer. The tubes were then directly gassed with N_2_/CO_2_ using a Hungate needle, sealed airtight with the butyl stopper and incubated at 37 °C on a horizontal shaker (PSU-20i, bioSan, Riga, Latvia, 120 rpm). The mixed preculture with the multiple species was centrifuged at 13,780 × g and frozen to later determine the exact mixing ratio using real-time PCR. Further samples of the growing cultures were taken after 12 or 24 h. For this purpose, the side of the butyl stopper was sterilized with 70% ethanol, and the alcohol was flamed off. The samples were taken with a sterile 1-mL disposable syringe and a small sterile disposable cannula by turning the culture tube upside down and inserting the cannula through the sterile area of the stopper. To prevent air from entering the culture tube, the syringe was first flushed several times with N_2_ via a Hungate needle and gas was drawn up during the last rinse with N_2._ When the sample was taken, N_2_ was pressed into the culture tube to counteract the negative pressure that could arise when taking the samples. Samples (0.2 mL) were taken, and the OD was determined. If the OD was above 1, the cell suspension was diluted with sterile medium, and the OD of the dilution was measured. After 36 h, the stopper was removed, a sample was taken for OD determination and pH measurement, and the remainder was centrifuged and frozen to determine the cell number after DNA extraction. The supernatant was used for analysis of organic acids.

**TABLE 1:** Species and substrate combinations in the growth trials.

*B. species*	Substrates and substrate combinations							
*B. l.* sub. *infantis*	2’FL (5)	6’SL (5)	2’FL/6’SL (5)	2’FL/6’SL (10)	2’FL/Lac (10)	Glc (5)	Lac (5)	Without substrate
*B. breve*	2’FL (5)	6’SL (5)	2’FL/6’SL (5)	2’FL/6’SL (10)	2’FL/Lac (10)	Glc (5)	Lac (5)	Without substrate
*B. bifidum* JCM 7004	2’FL (5)	6’SL (5)	2’FL/6’SL (5)	2’FL/6’SL (10)	2’FL/Lac (10)	Glc (5)	Lac (5)	Without substrate
*B. bifidum* JCM 1254	2’FL (5)	6’SL (5)	2’FL/6’SL (5)	2’FL/6’SL (10)	2’FL/Lac (10)	Glc (5)	Lac (5)	Without substrate
*B. inf.* + *B. breve*	2’FL (5)	6’SL (5)	2’FL/6’SL (5)	2’FL/6’SL (10)	2’FL/Lac (10)	Glc (5)	Lac (5)	Without substrate
*B. inf.* + *B. bifidum*	2’FL (5)	6’SL (5)	2’FL/6’SL (5)	2’FL/6’SL (10)	2’FL/Lac (10)	Glc (5)	Lac (5)	Without substrate
*B. breve* + *B. bifidum*	2’FL (5)	6’SL (5)	2’FL/6’SL (5)	2’FL/6’SL (10)	2’FL/Lac (10)	Glc (5)	Lac (5)	Without substrate
*B. inf. +* *B. breve +* *B. bifidum*	2’FL (5)	6’SL (5)	2’FL/6’SL (5)	2’FL/6’SL (10)	2’FL/Lac (10)	Glc (5)	Lac (5)	Without substrate

Concentrations of substrates are given as total concentrations in mM in parentheses. 2’FL, 2’fucosyllactose; 6’SL, 6’sialyllactose; Lac lactose; Glc, glucose. *B. bifidum* strain JCM 7004 was used in the growth experiments with several species.

### 2.4 Analysis of organic acids

Organic acids such as lactate and SCFA including acetate, propionate, isobutyrate, butyrate, isovalerate and valerate as well as formate and pyruvate were measured in supernatants of culture media before and after bacterial fermentation. High pH ion chromatography with suppressed conductivity on an ICS-5000 system (ThermoFisher, Dreieich, Germany) was used as described by Gotoh et al. (2017). Briefly, organic acids were isocratic separated on an IonPac AS11HC column (4 × 250 mm, ThermoFisher) using 1 mmol/L NaOH as mobile phase A and 100 mmol/L NaOH as mobile phase B at a flow rate of 0.75 mL/min, with a gradient starting at 23 min (0% B) to 33.3% B at 37 min, then 60% B at 47 min, after 2 min, mobile phase B was reduced to 0% again over 5 min and the column equilibrated for another 6 min. Total run time was 60 min, the ADRS 600 suppressor (4 mm, ThermoFisher) current was adjusted between 2 and 112 mA according to the mobile phase gradient. Samples were diluted before automated injection with mobile phase when appropriate, external validation and quantitation standards (Sigma-Aldrich, Steinheim, Germany) were part of the injection queue at regular intervals with upper quantitation limits of about 90 mM for lactate, acetate and isobutyrate, 30 mM for other compounds; 8 standard points were used for the higher concentrations (90 mM), 4 for the lower concentrated organic acids (30 mM). The Chromeleon 7.3 Software (ThermoFisher) was used, data processing was done with Microsoft Excel 365.

### 2.5 Statistics

Data were described as mean and standard deviations. Statistical tests were carried out using robust non-parametric median tests. Given the low power of the tests, due to the small sample size (*n* = 3), no correction was applied for the potential accumulation of alpha errors following multiple testing. The results of the significance tests should be interpreted as exploratory. The tests were carried out using IBM SPSS Statistics 26.

The heatmap and the correlation analysis of the OD values against the total organic acid concentrations were calculated and visualized with R version 4.4.2 (R Core Team, 2024) in R-studio v2024.09.1 with the additional R packages ggplot v3.5.2 (Wickham, 2016), readxl v.1.4.5 (Wickham and Bryan, 2025), dplyr (Wickham et al., 2025a), tidyr (Wickham et al., 2025b), and ComplexHeatmap (Gu, 2022).

## 3 Results

### 3.1 Growth of *Bifidobacterium* strains in various carbohydrate substrates

**FIGURE 1:**
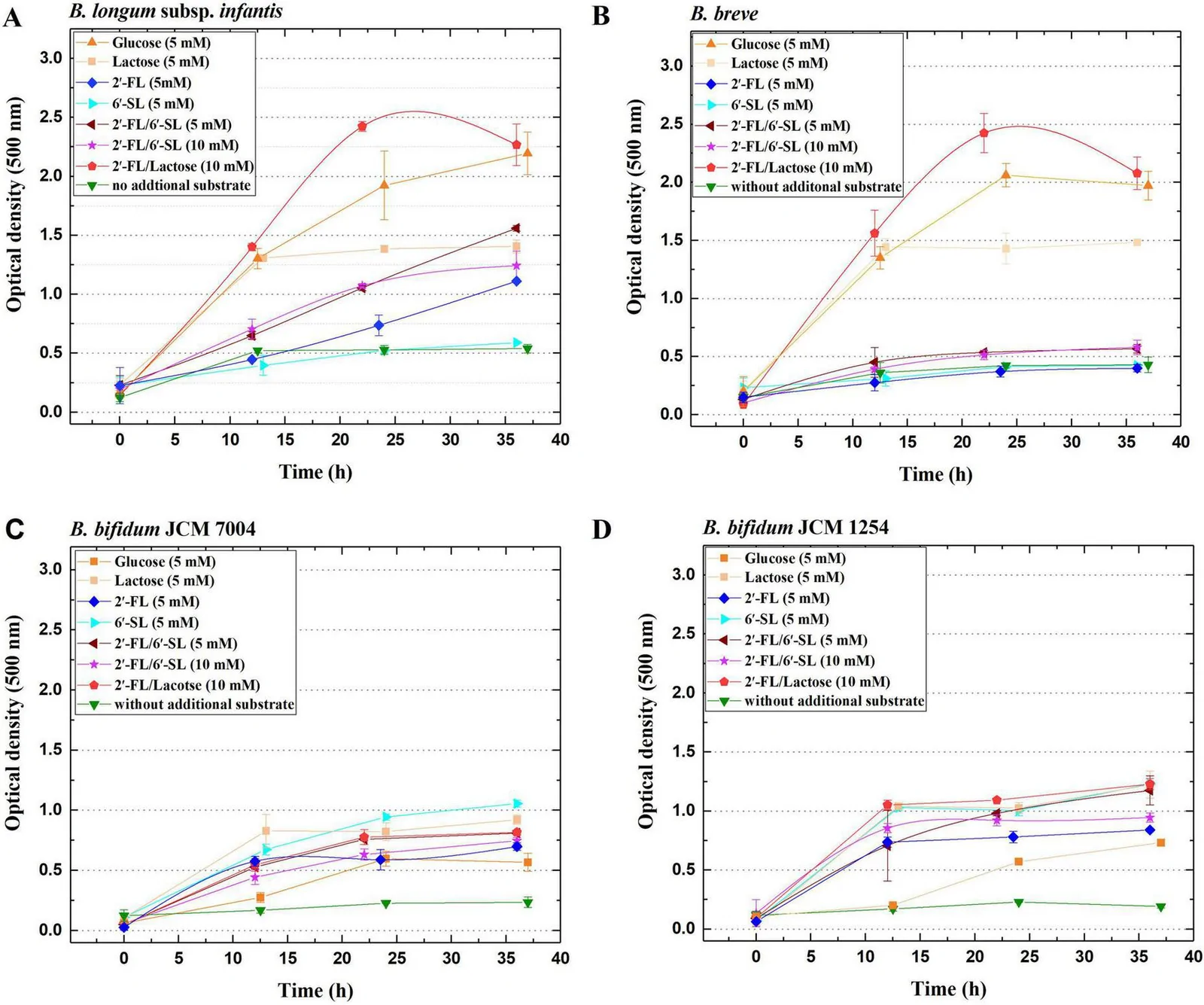
Optical density (at 500 nm) of anaerobic growth of *Bifidobacterium* sp. with **(A)** *B. longum* subsp. *infantis*, **(B)** *B. breve*, **(C)** *B. bifidum* JCM 7004, and **(D)** *B. bifidum* JCM 1254 in medium without carbohydrate addition or supplemented with 2’-FL and 6’-SL as well as glucose and lactose as controls and combinations thereof during 36 h incubation. Mean values (*n* = 3) and standard deviations are shown.

Growth experiments with single *Bifidobacterium* species using 2’-FL and 6’-SL as carbohydrate substrates or lactose and glucose as controls, as well as carbohydrate mixtures showed clear differences between the species (Figure 1). *B. longum* subsp. *infantis* and *B. breve* revealed the highest OD values with glucose (5 mM) and the combination of 2’-FL and lactose (10 mM). These values were significantly higher than the OD values for the other substrates (*p* = 0.014) (Supplementary material S2). With lactose, growth stopped after 12 h in both species and the OD remained at the same level (Figures 1A,B). While *B. breve* did not grow well with any other substrates, the OD in *B. longum* subsp. *infantis* cultures with 2’-FL combined with 6’-SL (5 and 10 mM) increased during the whole incubation time and reached a similar OD as in the experiment with lactose after 36 h. When 2’-FL was provided as the only substrate, bacterial growth was slower, as indicated by a more gradual increase in OD, and the final OD was slightly lower than that observed with 2′-FL/6′-SL or lactose after incubation. 6’-SL alone did not support the growth of *B. longum* subsp. *infantis*, as the same growth was observed in the control culture without additional carbohydrates (Figure 1A). For the two *B. bifidum* strains, no major differences were observed with the different carbohydrate substrates although *B. bifidum* JCM 7004 derived from infant gut grew slower than *B. bifidum* JCM 1254, a strain originally isolated from adult intestine. Compared to the other two *Bifidobacterium* species, the OD remained much lower for all substrates including the control without additional substrate. In general, the highest OD with 6’-SL (5 mM) was observed for the infant strain of *B. bifidum* JCM 7004 (*p* = 0.014) (Supplementary material S2) and the lowest with glucose as carbohydrate substrate (Figure 1C), whereby growth with glucose (5 mM) during the first 12 h was very similar to that of the control without additional carbohydrate. For the adult *B. bifidum* strain JCM 1254, the highest OD was found with the substrates 2’-FL/lactose (10 mM in total), 6’-SL (5 mM) and lactose (5 mM) and the lowest with glucose (5 mM) (Figure 1D).

**FIGURE 2:**
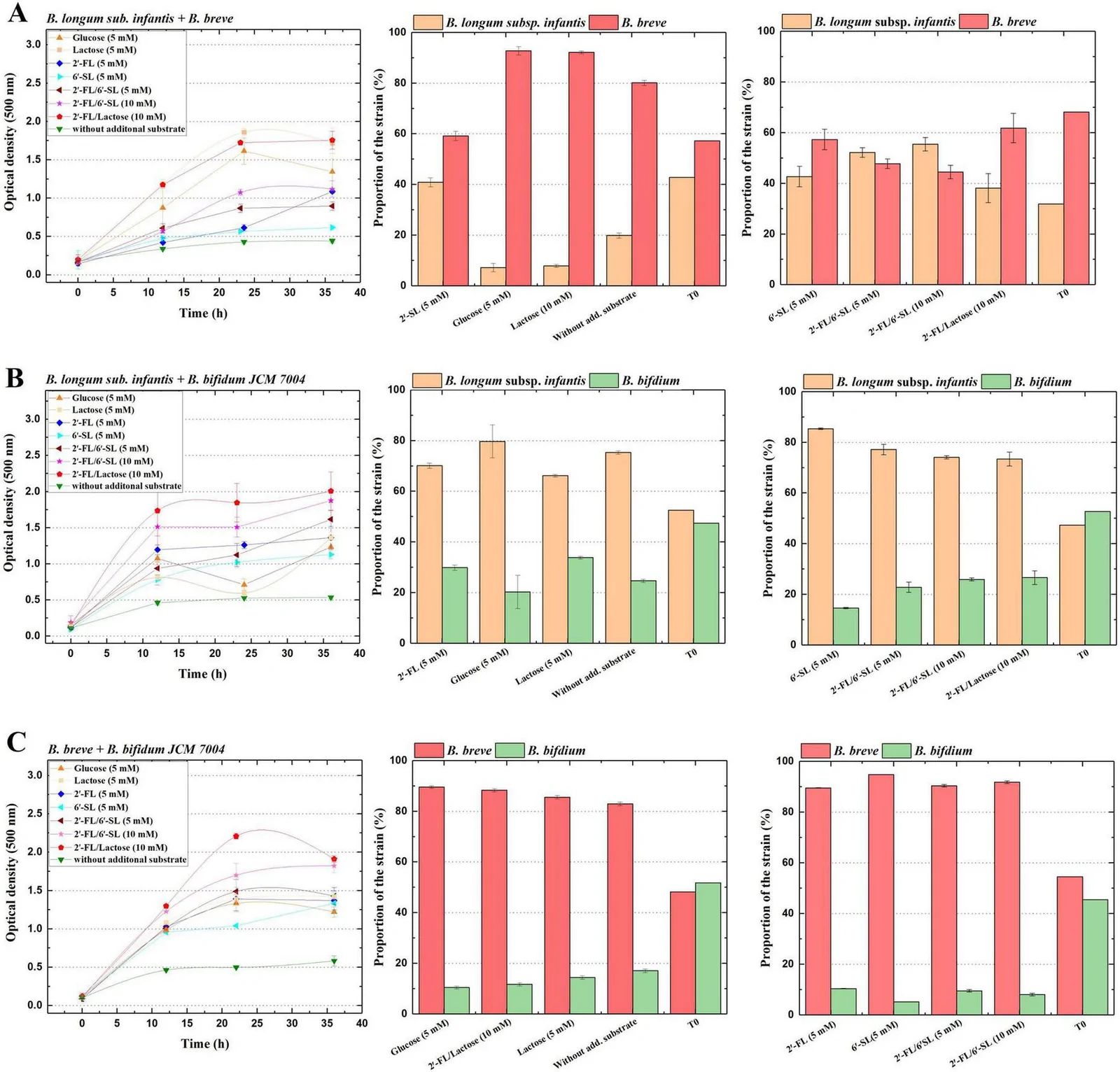
Optical density (at 500 nm) of anaerobic growth of *Bifidobacterium* sp. combinations. **(A)** *B. longum* subsp. *infantis*/B. *breve*, **(B)** *B. longum* subsp. *infantis*/*B. bifidum* JCM 7004, and **(C)** *B.* breve/*B. bifidum* JCM 7004 with 2’-FL and 6’-SL and controls (glucose, lactose, without additional carbohydrate substrate) and combinations thereof during 36 h incubation. The adjacent bar graphs show the percentage of *Bifidobacterium* species after 36 h with the respective substrate. The bars labeled T0 indicate the percentage of species in the inoculation culture. Shown are mean values (*n* = 3) and the standard deviations; T0 value (*n* = 1).

In the growth experiments with 2 species, which were inoculated with approximately the same total number of bacteria (Supplementary Table S1.1), the cell number was also determined after 36 h. The same substrates were used as in experiments with single species. In the mixture of *B. longum* subsp. *infantis* with *B. breve* (Figure 2A), the fastest increase and highest OD [significant ODs (*p* = 0.014), Supplementary material S2] were observed with the 2’-FL/lactose (10 mM) and lactose (5 mM) followed by glucose (5 mM). While *B. breve* clearly dominated cell count ratios after 36 h with glucose and lactose supplementation, this was not as pronounced with single HMOs (5 mM) or 2’-FL/lactose (10 mM) (Figure 2A). For the other substrates, a significant even higher OD (*p* = 0.014, Supplementary material S2) was observed for 2’-FL/6’-SL at 5 mM and 10 mM and for 2’-FL (5 mM) compared to the control without additional carbohydrate substrate. With the 5 mM supplementation of 2’-FL, the initial increase in OD was slow, similar to the control condition without carbohydrate supplementation or 6’-SL (5 mM) supplementation. However, from 24 h onwards, there was a stronger increase in OD which ultimately reached levels comparable to the culture with 2’-FL and 6’-SL at 10 mM (Figure 2A).

In all co-cultures of *Bifidobacterium* strains a subset of substrate conditions was able to support relatively high OD of about 2.0. This was not the case for single species cultures with *B. bifidum*. However, when *B. bifidum* was grown together with *B. longum* subsp. *infantis*, the substrate mixture 2’-FL/lactose (10 mM) produced the highest OD and the fastest increase in OD. In contrast to the co-culture of *B. longum* subsp. *infantis* and *B. breve* (Figure 2A), the mixture of 2’-FL with 6’-SL in the medium had similar growth effects to 2’-FL/lactose when *B. bifidum* was co-cultured with *B. longum* subsp. *infantis* (Figure 2B). Cultures with other substrates led to similar OD. After 36 h, the culture with the mixture of 2’-FL and 6’-SL (5 mM), 2’-FL and 6’-SL (10 mM) and 2’-FL/lactose (10 mM) had the highest OD and the culture with 6’-SL only (5 mM) had the lowest OD. All cultures with carbohydrate supplementation in the medium had grown significantly (*p* = 0.014, Supplementary material S2) better after 36 h compared to the control cultures without additional substrate. *B. longum* subsp. *infantis* always dominated in mixtures with *B. bifidum* after 36 h (Figure 2B). Very similar to *B. longum* subsp. *infantis* and *B. bifidum* was the development of the co-culture of *B. breve* and *B. bifidum* (Figure 2C). Cultures with the carbohydrate mixture 2’-FL and lactose (10 mM) produced the highest OD, followed by the culture with the mixture of 2’-FL and 6’-SL (10 mM). Cultures with other substrates were similar and significantly different from the OD of the culture without additional substrate (*p* = 0.014, Supplementary material S2). Quantitative real-time PCR showed that in co-cultures of *B. breve* with *B. bifidum*, the ratio of the two strains was about 9:1. The proportion of *B. bifidum* was always low and independent of the OD representing the growth of the co-culture (Figure 2C).

**FIGURE 3:**
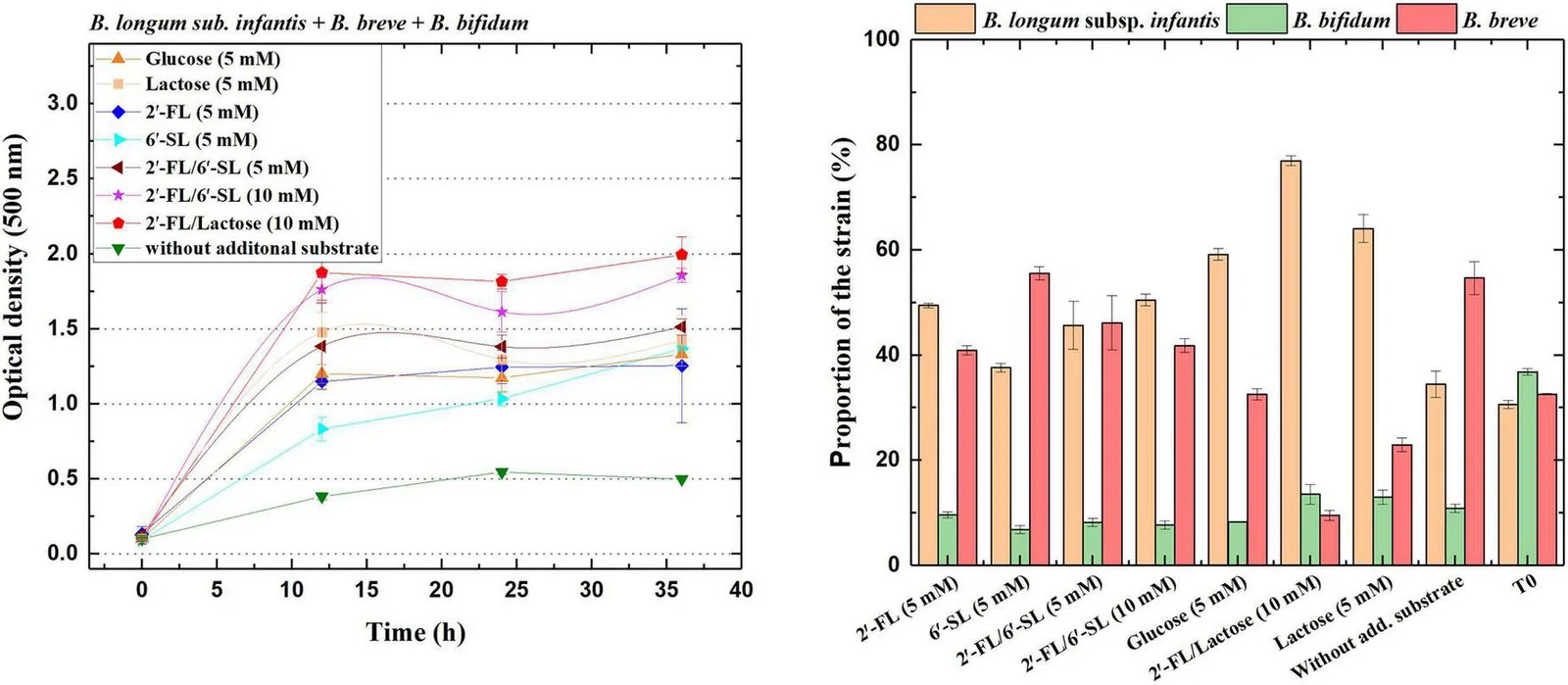
Optical density (at 500 nm) of anaerobic growth of *B. longum* subsp. *infantis*, *B. breve* and *B. bifidum* JCM 7004 with 2’-FL and 6’-SL and controls (glucose, lactose, without additional carbohydrate substrate) and combinations thereof during 36 h of incubation. The adjacent bar graphs are the percentage of *Bifidobacterium* species after 36 h with the respective substrate. The bars labeled T0 indicate the percentage of species in the inoculation culture. Mean values (*n* = 3) and standard deviations are shown.

In the growth experiment with all 3 *Bifidobacterium* species (Figure 3), the growth curves represented by the increase in OD were similar to what has been observed for the co-cultures with *B. bifidum* described above (Figures 2B,C). Again, the cultures with the substrate mixtures 2’-FL/lactose and 2’-FL/6’-SL at the high concentrations (10 mM) showed the highest OD (*p* = 0.014, Supplementary material S2) and the cultures with the other substrates had a very similar growth pattern compared to the cultures with 6’-SL (5 mM) showing a slightly lower OD at the beginning. Cultures without additional substrate revealed the lowest OD (*p* = 0.014, Supplementary material S2). With regard to the ratios between the 3 strains, the ratio between *B. longum* subsp. *infantis* and *B. breve* was balanced for the substrates 2’-FL (5 mM), 2’-FL/6’-SL with 5 and 10 mM, while *B. longum* subsp. *infantis* dominated when glucose (5 mM) was supplemented and even more pronounced when lactose (5 mM) or 2’-FL/lactose (10 mM) were present in the media. In contrast, *B. breve* was found more frequently in cultures with 6’-SL (5 mM) and in cultures without carbohydrate substrate; these, however, had the lowest growth (Figure 3). Regardless of the substrate supplemented, *B. bifidum* was the least abundant strain in all cultures after 36 h.

When *B. bifidum* was grown in co-culture with 6’-SL-supplemented media, the *Bifidobacterium* mix showed good growth, although only half or one third of the initial bacteria count represented *B. bifidum* in the 2-strain and 3-strain mix, respectively. Although *B. bifidum* was the only strain capable of utilizing 6′-SL in monoculture, it was outcompeted in co-culture by *B. longum* subsp. *infantis*, which accounted for approximately 85% of the bacterial population, and even more so by *B. breve*, which represented about 95% of the total bacterial counts as determined by quantitative real-time PCR (qPCR) (Figures 2B,C). When all three strains were cultured together, growth on 6′-SL-supplemented medium resulted in the highest OD values observed among all single- and mixed-culture experiments. However, *B. bifidum* represented only approximately 5% of the total bacterial population, whereas *B. longum* subsp. *infantis* and *B. breve* accounted for about 40 and 55%, of the total population, respectively (Figure 3).

### 3.2 Effect of carbohydrate supplementation on the pH of the bacterial media

As expected, the pH values after 36 h correlated with the OD values. The stronger the growth, the lower the pH value (Table 2). The highest pH values were found in cultures without additional substrate and in those that had not grown despite carbohydrate supplementation. However, looking at the dimension of growth, *B. bifidum* regularly produced low pH (<5.00) levels on any carbohydrate supplement although its lower OD values compared to all other *Bifidobacterium* strains tested (Table 2). With 2’-FL/lactose supplementation, the medium of all strains had reached a low pH value after 36 h, and with 2’-FL/6’-SL, only the culture with *B. breve* as single strain culture did not.

**TABLE 2:** pH-value [mean (*n* = 3) ± SD] of the culture media dependent on substrate supplement.

*B. strains*	No add. substrate	Glc (5)	Lac (5)	2’-FL (5)	6’-SL (5)	2’-FL/6’-SL (5)	2’-FL/6’-SL (10)	2’-FL/Lac (10)
*B. l. sub. infantis*	6.24 ± 0.09	4.80 ± 0.03	4.75 ± 0.07	4.50 ± 0.00	5.80 ± 0.02	4.62 ± 0.05	4.43 ± 0.01	4.37 ± 0.05
*B. breve*	6.42 ± 0.01	4.78 ± 0.04	4.55 ± 0.01	6.05 ± 0.15	6.15 ± 0.03	6.23 ± 0.02	5.94 ± 0.35	4.39 ± 0.03
*B. bifidum* (JCM 7004)	6.41 ± 0.01	4.98 ± 0.01	4.71 ± 0.03	4.77 ± 0.01	4.64 ± 0.02	4.70 ± 0.10	4.58 ± 0.09	4.48 ± 0.03
*B. bifidum* (JCM 1254)	6.35 ± 0.06	4.94 ± 0.04	4.64 ± 0.03	4.71 ± 0.01	4.77 ± 0.13	4.59 ± 0.03	4.48 ± 0.01	4.41 ± 0.06
*B. l. sub. infantis* *+ B. breve*	6.19 ± 0.05	4.70 ± 0.12	4.47 ± 0.06	4.53 ± 0.01	6.23 ± 0.19	5.81 ± 0.12	5.03 ± 0.23	4.75 ± 0.04
*B. l. sub. infantis + B. bifidum*	6.38 ± 0.02	5.45 ± 0.09	5.37 ± 0.06	5.28 ± 0.10	4.77 ± 0.06	4.81 ± 0.16	4.52 ± 0.03	4.54 ± 0.07
*B. breve* *+B. bifidum*	6.25 ± 0.12	5.70 ± 0.05	5.32 ± 0.04	5.40 ± 0.06	5.15 ± 0.03	5.14 ± 0.03	4.69 ± 0.08	4.78 ± 0.09
*B. l. sub. infantis* *+ B. breve* *+ B. bifidum*	6.37 ± 0.11	5.83 ± 0.01	5.32 ± 0.11	5.38 ± 0.02	5.15 ± 0.01	5.00 ± 0.07	4.75 ± 0.02	4.75 ± 0.02

2’-FL, 2’-Fucosyllactose; 6’-SL, 6’-Sialyllactose; Lac, Lactose; Glc, Glucose; no add. sub., no additional substrate. The *B. bifidum* strain JCM 7004 was used in the growth experiments with several species. Concentrations of substrates are given as mM in parentheses.

### 3.4 Organic acid production and growth

**FIGURE 4:**
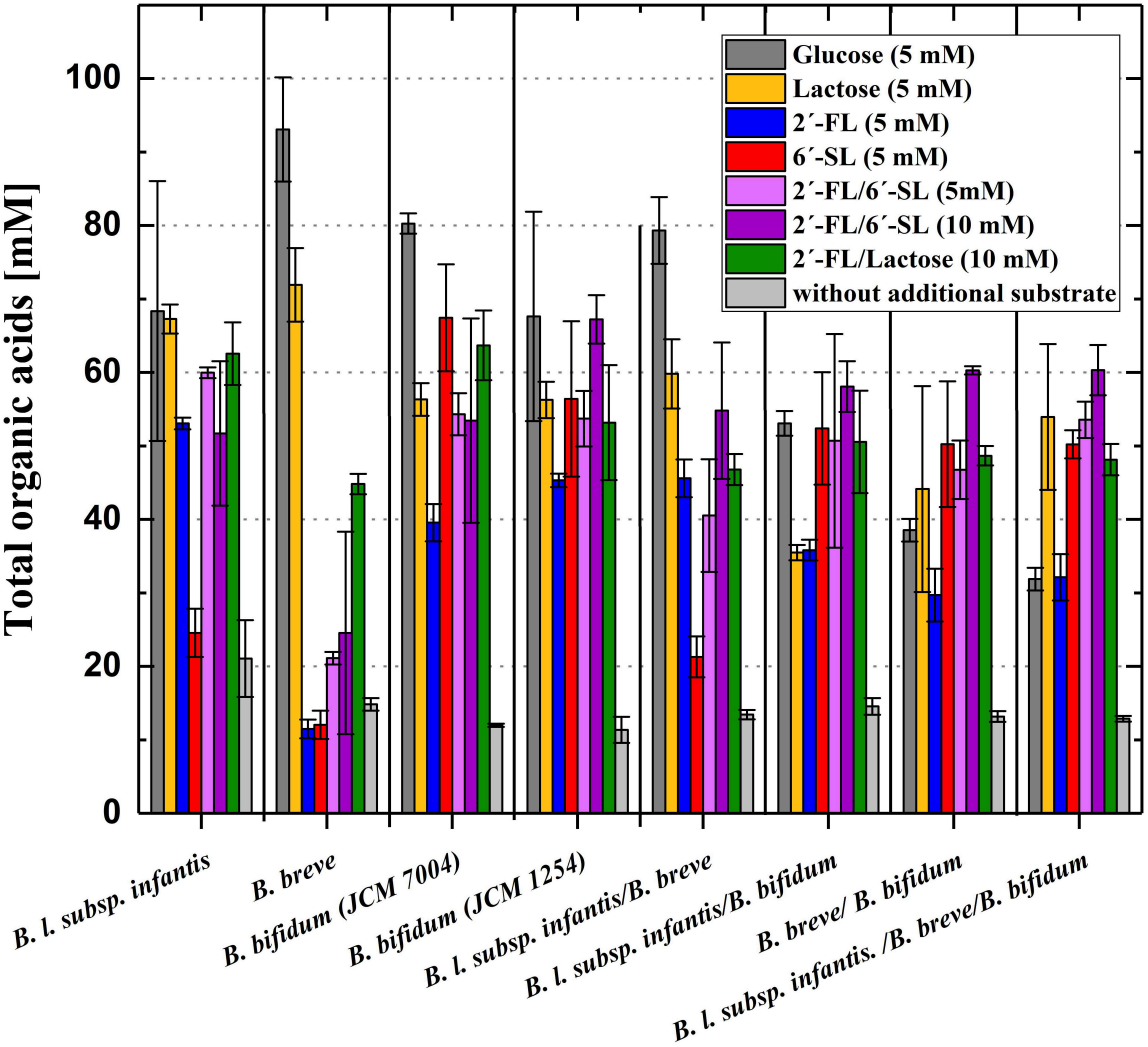
Total organic acids (in mM) in the various combinations of bacteria and substrates.

**FIGURE 5:**
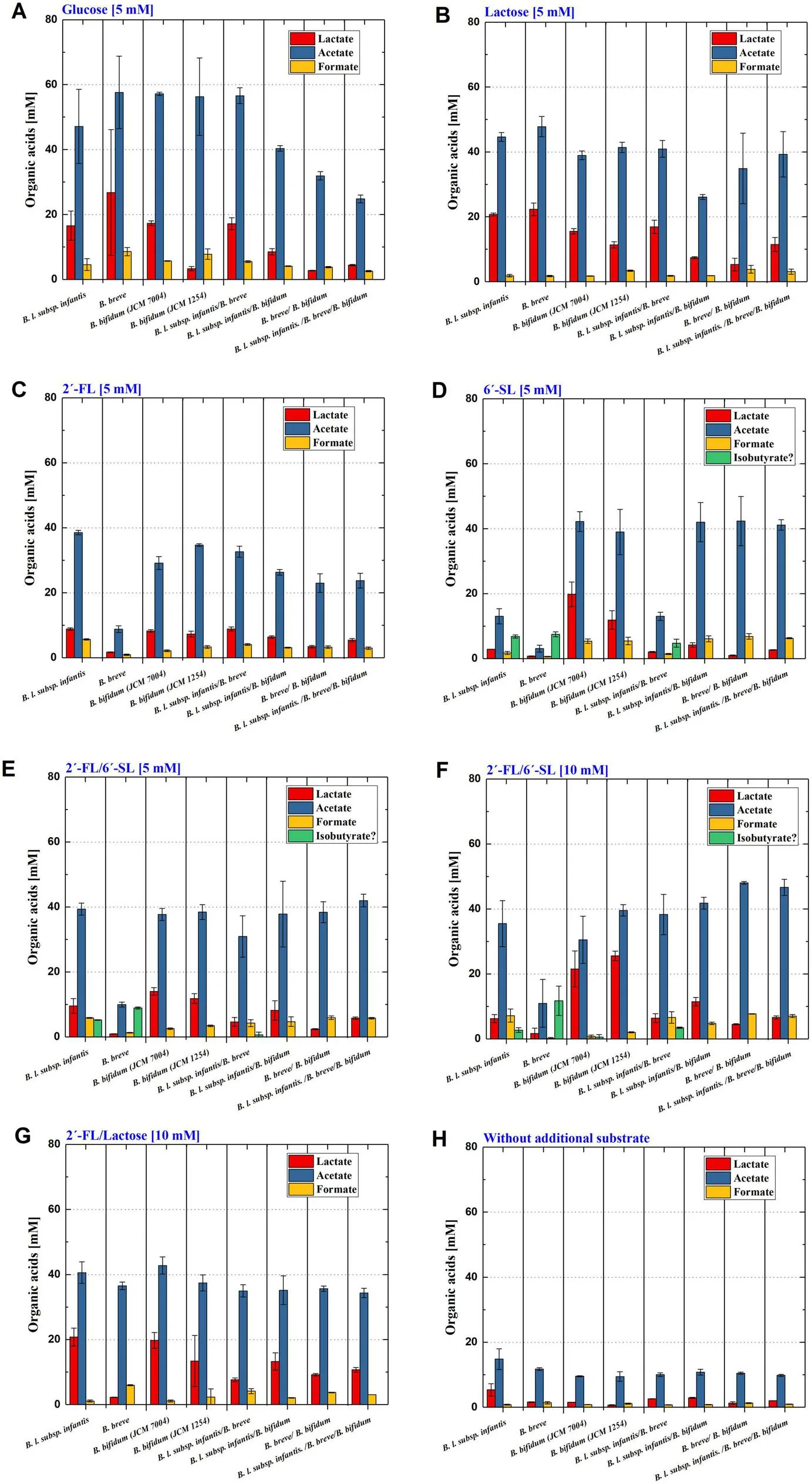
Concentration of organic acids (in mM) in the medium after 36 h incubation with *Bifidobacterium* sp. or incubation of different combinations of *Bifidobacterium* sp. as a function of added substrates **(A–G)** or without additional substrate **(H)**.

Organic acid levels in the media before bacterial fermentation were minor revealing 0.57, 2.41 and 0.14 mM lactate, acetate and formate, respectively. Overall, the production of organic acids as shown in Figure 4 was dependent on the growth of the bacterial cultures. For *B. bifidum* in single cultures, the density of growth determined as OD at 36 h, however, did not predict the concentration of total organic acids (Supplementary material S1, Figure S1.4) nor the pattern of single organic acids in the culture media (Figures 5E,F).

In mixed cultures with *B. bifidum*, the growth determined as OD did correlate with the production of total organic acids; the growth of *B. bifidum* within the culture, however, as determined by qPCR did not (Figures 2, 3).

Growth of *Bifidobacterium* strains was similar with lactose and glucose as positive controls for carbohydrate supplementation in the culture media, the production of organic acids differed markedly with concentrations of total organic acids of 80 mM and more in some of the cultures grown in glucose and about 60 mM for those cultured in lactose supplemented media with the exception of the mix of all three strains where lactose supplementation had favored the production of organic acids (Figure 4).

Comparing the two HMOs, 2’-FL and 6’-SL at equimolar carbohydrate supplementation in the culture media, the ability of the *Bifidobacterium* strains to grow on these substrates determined whether organic acids of more than about 50 mM were produced. The growth of *B. longum* subsp. *infantis* but not of *B. breve* was supported by 2’-FL leading to a high output of organic acids of about 50 mM and only low concentrations (comparable to concentrations in the controls without additional carbohydrates) in the media, respectively (Figure 4). Similar observations were made for *B. longum* subsp. *infantis* and *B. breve* which did not grow in 6’-SL supplemented media and only produced low amounts of organic acids. *B. bifidum* and co-cultures with other *Bifidobacterium* strains, however, grew well, revealing high concentrations of total organic acids which increased significantly from 32.1 ± 3.2 to 50.2 ± 1.9 mM (*p* = 0.014, Supplementary material S2). Despite the high output of organic acids in these co-cultures, the strains that did not grow in 6’-SL supplemented media as single strain cultures increased in cell numbers within these co-cultures and finally outnumbered *B. bifidum* (Figures 2–4). For the combination of 2’-FL/6’-SL (5 mM) in the media, apart from *B. breve*, all *Bifidobacterium* cultures were observed to grow well and to produce organic acids up to concentrations of more than 40–60 mM (for *B. longum* subsp. *infantis* as single strain) (Figure 4).

When comparing growth and output of total organic acids at the highest carbohydrate supplementation of the media (10 mM) as mixtures of 2’-FL/6’-SL or 2’-FL/lactose, the co-culture containing *B. bifidum* were found to grow similarly. The output of organic acids increased even more with 2’-FL/6’-SL than that revealed from the combination of 2’-FL and lactose with 60.3 ± 3.4 mM and 48.1 ± 2.1 mM, respectively (Figure 4). All *Bifidobacterium* strains and co-cultures thereof except for *B. bifidum* JCM1254 grew best in 2’-FL/lactose supplementation (Figures 1–3) and produced organic acids in concentrations of up to 60 mM (Figure 4).

Overall, glucose supplementation of the media led to the highest output of total organic acids, followed by the supplementation of lactose. When *B. bifidum* was present in single or co-cultures, 6’-SL alone or in combination with 2’-FL as well as 2’-FL with lactose revealed to be well-suited substrates for a high output of organic acids.

In media without carbohydrate supplementation, all ODs determined after 36 h remained low accompanied by a low production of organic acids (Figure 4).

### 3.5 Production of individual organic acids

Organic acids such as lactate, acetate and formate were found in all bacterial supernatants (Figures 5A–H), whereas valerate, isovalerate and pyruvate were detected in trace amounts only and subsequently excluded from further analysis. Supernatants from *Bifidobacterium* cultures grown in media without added carbohydrates contained only low concentrations of lactate, acetate, and formate. For *B. longum* subsp. *infantis*, the respective concentrations were approximately 5, 15, and 1 mM, whereas all other cultures produced significantly lower amounts of these organic acids. These findings indicate that a small proportion of the organic acids produced originated from the fermentation of amino acids and other components present in the medium, despite the minimal bacterial growth observed under these conditions (Figures 1–3, 5H). The concentrations of organic acids in non-inocculated medium were low with about 1 and 2 mM of lactate and acetate, respectively; formate was found even below 1 mM.

In carbohydrate supplemented media, formate was found consistently (Figures 5A–G), but mostly in low amounts up to 5 mM; the highest concentrations up to 8 mM were observed in bacterial supernatants from *B. breve* and *B. bifidum* JCM 1254 when grown with glucose supplementation of the media which was used as positive control for *Bifidobacterium* growth.

With glucose supplementation of the media, high acetate and lactate production was reached with mean concentrations up to about 60 and 30 mM, respectively (Figure 5A). Glucose was the substrate leading to the highest output of acetate in single strain cultures and co-cultures of *B. longum* subsp. *infantis* and *B. breve* (Figure 5B).

Lactate was produced up to mean concentrations of about 10 to more than 20 mM by all *Bifidobacterium* strains dependent on various substrates, not only when lactose as a physiological control was present in the media (Figures 5B–G).

Using 2’-FL as carbohydrate supplement, the single strain culture of *B. longum* subsp. *infantis* produced the highest concentrations of acetate with 38.6 ± 0.7 mM; the lowest concentrations were found for *B. breve* (8.4 ± 1.0 mM) which, however, did not grow in 2’-FL supplemented media (Figure 5C).

When 6’-SL was supplemented to the media in equimolar concentrations (Figure 5D), single *Bifidobacterium* strains followed the pattern mentioned above, i.e., *B. longum* subsp. *infantis* and *B. breve* as well as their co-culture grew minimally and thus produced trace amounts of acetate and lactate. In contrast, both *B. bifidum* strains grew well on 6′-SL, resulting in high concentrations of acetate (approximately 40 mM) and lactate (up to 20 mM), indicating efficient utilization of this substrate. In co-culture, however, *B. bifidum* produced equally high acetate but low lactate concentrations concomitant with *B. bifidum* being outnumbered by the other *Bifidobacterium* strains when in co-culture (Figures 2, 3, 5D). Notably, in media containing 6′-SL, both *B. longum* subsp. *infantis* and *B. breve* produced an organic acid that, based on its retention time during HPLC analysis, was tentatively identified as isobutyrate. The same observation was made when these two strains were cultivated in co-culture, where the concentration of this compound reached approximately 10 mM (Figure 5D–F).

*Bifidobacterium* strains grown in media supplemented with a mixture of 2’-FL and 6’-SL (at 5 mM total), acetate was produced in all cultures in concentrations of about 40 mM except for *B. breve* which did not grow in these media (Figure 5E). At 10 mM 2’-FL/6’-SL, the production of organic acids further increased in co-cultures (Figure 5F), in single cultures of *B. longum* subsp. *infantis* and *B. breve*, however, the production of organic acids remained similar whereas in *B. bifidum* cultures, lactate production was found to be significantly increased (Figures 5E,F).

The most favorable growth conditions, with the exception of *B. bifidum* JCM 7004 were found for cultures in media supplemented with 2’-FL and lactose at a total concentration of 10 mM. Lactate concentrations between 2 and 20 mM were found in bacterial supernatants for *B. breve* and *B. longum* subsp. *infantis* or *B. bifidum* JCM 7004, respectively. Acetate production was high in all cultures with about 40 mM (Figure 5G).

Metabolic conditions of bacterial cultures can be derived from the ratio of organic acids as major metabolites. High ratios of lactate to acetate of about 1–2 were found for several substrates and substrate combinations such as for *B. longum* subsp. *infantis* and *B. bifidum* in the presence of lactose in the media and for *B. bifidum* only also with 6’-SL in the media. *B. breve* only produced higher lactate to acetate ratios when glucose or lactose as single carbohydrates were supplemented to the media. In co-cultures, the ratio was mostly lower with high acetate concentrations in the supernatants (Figure 5).

## 4 Discussion

*Bifidobacterium* species are considered critical for the establishment of a health-promoting intestinal microbiota in infancy. Bifidobacteria can metabolize monosaccharides, disaccharides and oligosaccharides as well as other host- or diet-derived glycans due to a number of glycosyl hydrolases and transferases for extracellular hydrolysis or degradation after internalization. No strain has been shown to utilize all glycans (Xiao et al., 2024). The increase in cell density of bacterial cultures *in vitro* by optical density and *in vivo* by measurements of colony forming units (cfu), however, is mostly used as a measure to evaluate their metabolic activity *in vitro* and *in vivo*, thus overlooking the ability of certain *Bifidobacterium* strains to adapt to their environment, which was shown for *B. breve* (Ruiz-Moyano et al., 2013), or to establish cross-feeding relationships by sharing their metabolites derived from extracellular glycosidase activity as in *B. bifidum*, such as fucose, sialic acid and lactose (Lawson et al., 2020, Xiao et al., 2024). This cross-feeding phenomenon not only affects the growth of different bacterial strains but also has a significant impact on host health. Thus, the aim of this study was to perform controlled *in vitro* experiments using the *Bifidobacterium* strains that are characteristic of the infant gut, namely *B. longum* subsp. *infantis*, *B. breve* and *B. bifidum* in single cultures but also as co-cultures of two or all three strains together. These strains are known to utilize HMOs with different specificities. However, growth can also be induced with other carbohydrates such as glucose or lactose.

This study demonstrates that growth characteristics of infant-associated bifidobacteria in monoculture do not reliably predict their behavior in mixed communities. Instead, strain interactions strongly influenced substrate utilization, community composition, and fermentation outcomes. In particular, *B. bifidum* played a disproportionately important ecological role despite often reaching lower cell densities than *B. longum* subsp. *infantis* or *B. breve*. Our findings further show that organic acid production depended not only on the carbohydrate substrate provided but also on the presence of metabolically complementary strains capable of cross-feeding interactions. A central finding was the importance of cross-feeding during utilization of HMOs. When *B. breve*, for example, was grown in media supplemented with 2’-FL in a mid-physiological concentration range (5 mM), it did not grow better than in media without additional carbohydrates. In co-cultures, however, its cell numbers significantly increased, especially when cultured together with *B. bifidum*. This is in agreement with observations by Walsh et al. (2022) who also found that *B. breve* which did not significantly grow on 2’-FL when used as single culture but showed increased cell numbers when cultured together with *B. longum* subsp. *infantis* and *B. bifidum*. Similar observations were made for bifidobacteria in 6’-SL-supplemented media in our study. The effects of cross-feeding were even more apparent during growth on 6′-SL. In single cultures, *B. bifidum* was the only strain which used 6’-SL for growth whereas *B. longum* subsp. *infantis* and *B. breve* did not (Figure 1). In co-cultures, however, both strains benefited from the presence of *B. bifidum*, resulting in markedly enhanced growth (Figures 2, 3). This may be supported by observations of Ruiz-Moyano et al. (2013) who found that various phenotypes of *B. breve* displayed specific adaptations to carbohydrate substrates, which may help to explain the predominance of this strain in breast-fed infant feces.

This observation is consistent with the specialized extracellular glycosidase activity of *B. bifidum*, *which* express a complete set of membrane-bound glycosyl hydrolases including α-sialidase and α-fucosidase for the cleavage of O-glycans such as HMOs on the outside of the cell membrane (Nishiyama et al., 2018; Lawson et al., 2020; Katoh et al., 2020). Through extracellular hydrolysis, metabolites become available to neighboring bacteria and support the growth of strains that are otherwise unable to efficiently access the original substrate. The observations above may be consistent with prior reports that *B. bifidum* can hydrolyze 6’-SL by cleaving off sialic acid outside the cell, which makes it available to other strains in co-cultures (Nishiyama et al., 2018). For other bifidobacterial species such as *B. breve* and *B. longum* subsp. *infantis*, gene clusters have been identified to internalize and metabolize sialic acid to pyruvate and N-acetyl-mannosamine which can be further processed via N-acetyl-glucosamine to glutamine, glutamate and acetyl-CoA (Ward et al., 2007; Egan et al., 2014; Li et al., 2023). The generation of amino acids during the metabolism of sialic acid may thus explain the growth advantage of *B. longum* subsp. *infantis* and *B. breve* when cultured in the presence of a nitrogen containing HMOs such as 6’-SL. In addition, these *Bifidobacterium* strains also compete with *B. bifidum* for lactose and finally outnumber it due to their faster growth rates on this substrate.

In the presence of 2’-FL, the situation for single versus mixed cultures with *B. bifidum* is similar. However, here, *B. longum* subsp. *infantis* grows well in 2’-FL-supplemented media even as single strain (Figure 1). When grown together with other bifidobacteria, the rapid utilization of 2’-FL provides benefits for *B. longum* subsp. *infantis* over *B. bifidum*, but not over *B. breve* (Figure 2). In the presence of all three strains, *B. longum* subsp. *infantis* has some advantages in 2’-FL-supplemented media, which were found to be enhanced when lactose was added to the media (Figure 3). These observations show that the growth determined as an increase in OD in single strain cultures would not be an indicator of the ability of a strain to take advantage when grown in co-culture, i.e., growth behavior of a single strain culture does not predict which strain benefits most in mixed cultures of more than two strains. Accurate growth evaluation of each *Bifidobacterium* strain especially in co-cultures is only possible using specific qPCR assays for each strain (Figures 2, 3).

The ability of bifidobacteria to utilize 2‘- or 3-FL by either internalization via ABC transporters and subsequent degradation to acetate and other organic acids as has been shown for *B. longum* subsp. *infantis* or extracellular fucosidase activity in *B. bifidum* cleaving 2’-FL into fucose and lactose providing carbohydrates also to other bifidobacterial strains for growth and metabolism (Matsuki et al., 2016; Centanni et al., 2019; Sakanaka et al., 2019). This highlights the importance of the co-existence of various strains and their cross-feeding effects, which result in differential acid production and health benefits.

Lactose itself, the backbone of the HMO structure, also supports bifidobacteria growth, although not in a selective way, but as a carbohydrate which can be used for multiple bacterial species. Here, only 5 mM of lactose were used as a supplement for the growth media either as a single carbohydrate or in combination with 2’-FL. This amount is equivalent to about 2% of the total lactose content in breast milk which may well escape digestion and reach the lower parts of the intestine with high bacterial colonization. When lactose was supplemented to the culture media either as sole carbohydrate or in combination with 2’-FL, lactate was produced in concentrations up to 20 mM (Figure 5). These lactate concentrations were also reached with glucose supplementation of the media; however, glucose was used as a positive control for optimal bacterial growth *in vitro*. The amount of 5 mM free glucose as substrate for bifidobacteria does not represent the *in vivo* situation, since glucose would have been absorbed in the human gut before reaching the intestinal microbiota.

In the presence of 2’-FL, however, acetate was found to be a major metabolite, and the overall production of organic acids was significantly lower than with lactose. Here, *B. bifidum* already showed its high capacity for acid production. Although its growth after 36 h only reached about an OD of 0.7, its production of organic acids revealed pH levels as low as those from *B. longum* subsp. *infantis* with a markedly higher OD of 1.2 and still rising at that time (Figure 1 and Table 2). Zabel et al. (2019) concluded that 2’-FL metabolism in *B. longum* subsp. *infantis* is a complex process involving multiple gene clusters that produce a more diverse metabolic profile compared to lactose which confirmed the production of formate, acetate, 1,2-propanediol and lactate and other intermediate products such as pyruvate which can be further metabolized to acetate and formate. The pattern of organic acid we observed for *B. longum* subsp. *infantis* metabolizing lactose or 2’-FL was similar, the concentrations of lactate and acetate, however, were significantly higher with lactose in the media. This may be due to the different strain we used or to a later time point during the growth phase, when intermediate products have already been further metabolized by the bacteria.

With the addition of further carbohydrates to the media, the output of organic acids also increased for all *Bifidobacterium* strains except for *B. breve* which required lactose supplementation or the presence of other *Bifidobacterium* strains mainly *B. bifidum* known for its cross-feeding ability mentioned above (Lawson et al., 2020; Xiao et al., 2024).

The best growth overall (OD about 2) was observed in media supplemented with the combination of 2’-FL and lactose (10 mM). This was true for all strains and combinations of strains except for *B. bifidum* as single strain. Here, an OD of only about 0.7–1 was reached (Figures 1–3). Nevertheless, 2’-FL and lactose promoted consistently high acid production even in *B. bifidum* where growth did not correlate with organic acid production (Supplementary Figure S1.4).

In addition, the presence of *B. bifidum* was the prerequisite for mixed cultures to grow on 6’-SL above an OD of 1. Although *B. bifidum* was outnumbered by *B. longum* subsp. *infantis* and *B. breve*, its presence was responsible for the production of high amounts of organic acids mainly acetate.

Thus, the fermentation of 6’-SL in the presence of *B. bifidum* significantly changed when different strains were used in co-culture. Among the strains tested in monoculture, only *B. bifidum* was able to grow on 6′-SL and produced high concentrations of the organic acids lactate and acetate. In contrast, *B. longum* subsp. *infantis* and *B. breve* did not exhibit substantial growth in 6′-SL-supplemented medium. Nevertheless, an additional metabolite was detected in the culture supernatants of both strains that showed the same retention time as isobutyrate in the HPLC analysis, suggesting the possible formation of this compound. In the case of 6’-SL supplementation together with 2’-FL, both strains grew equally well in co-culture and continued to produce the hypothesized isobutyrate compound in addition to lactate and formate in small amounts. However, overall, acetate was the major acid metabolite produced. In mixed cultures with *B. bifidum*, acetate concentrations were even higher, but “isobutyrate” was missing. The presence of *B. bifidum* revealed efficient acid production as single strain with all carbohydrates or in mixed cultures with HMOs in the media explaining the lower pH levels reached by the combination of the two HMOs (Figure 4 and Table 2).

This may be an important observation with regard to the differential development of the intestinal microbiota. In breastfed infants, about 70–80% receive high amounts of 1–5 g/L 2’-FL due to the secretor status of the mother, i.e., the expression of FUT2 and the subsequent secretion of α1–2-fucosylated compounds such as 2’-FL in milk (Thurl et al., 2017). This may then promote the establishment of *B. longum* subsp. *infantis* which metabolizes 2’-FL to acetate and increased lactate production when lactose is present at the same time.

Thus, the metabolic utilization of the full set of glycosyl hydrolases found in *B. bifidum* (Xiao et al., 2024) seems to be adapted depending on the substrate present. The competition for substrates may up-regulate gene expression for enzymes and transporters involved in the utilization of different HMOs. As has previously been reported by others, (Gotoh et al., 2018; Centanni et al., 2019; Xiao et al., 2024), *B. bifidum* is a key species the microbiome due to its ability to degrade HMOs such as 6’-SL affecting community growth kinetics and providing resources for the rest of the consortium. Thus, *B. bifidum* facilitates cross-feeding and has a major impact on the production of organic acids although it may be outnumbered by other *Bifidobacterium* strains. This may be due to their faster metabolism and broader spectrum of transporters and intracellular enzymes to degrade HMO-derived monosaccharides such as fucose and sialic acid (Gotoh et al., 2018; Pröschle-Donoso et al., 2025). *B. bifidum* is found as a member of the intestinal microbiota over a wide age range although it is not a predominant strain. Instead, it may impact microbiota composition through cross-feeding of HMO or mucin degradation (Katoh et al., 2020).

The presence of *Bifidobacterium* strains such as *B. bifidum* with a high sialidase activity like the two strains we used in our study, were shown to enhance epithelial barrier integrity. This was reported by Segui-Perez et al. (2025) for two probiotic *B. bifidum* strains as shown in co-culture with human intestinal cell lines. *B. bifidum* was found to exhibit a notable genomic specialization toward the metabolism of host mucin O-glycans, HMOs and their degradation products as mentioned above. Other bifidobacteria were found to be specialized on HMO degradation, although their glycan utilization pathways may lack the *Nan* gene cluster encoding a well characterized sialic acid catabolic pathway (Arzamasov et al., 2025) which would explain the inability of the *B. longum* subsp. *infantis* and *B. breve* we used to metabolize 6’-SL in single or co-culture.

According to Ruiz-Moyano et al. (2013), *B. longum* subsp. *infantis* or *B. bifidum* have regularly been shown to utilize HMOs *in vitro* as the sole carbon source whereas *B. breve* isolates appear to be less adapted to these substrates. Considering the high frequency at which *B. breve* is isolated from breast-fed infant feces, they postulated that the HMO consumption type of *B. breve* is variable. By glycoprofiling the supernatants after 70 h of growth *in vitro*, they found that some strains showed specific adaptations to the substrates especially when HMOs were more complex.

In our study, the HMOs used were small structures consisting of only three monosaccharides but revealed high acidic production, especially when used in co-cultures together with *B. bifidum*. The production of high levels of acetate reveals efficient substrate metabolism and ATP production as well as an additional energy source for the infant (Kasubuchi et al., 2015; Kim and Kim, 2017; Kim, 2018; Hays et al., 2024; Bui et al., 2025). Most importantly, organic acids such as acetate or lactate have specific health effects apart from their pH lowering effect which creates a less favorable environment for pathogenic bacteria. Lactate could be further used as an energy substrate but may also serve as a signaling molecule in the brain (Cai et al., 2022).

In addition, acetate and propionate may enhance Th17 cell formation and increase the population of Th1 cells essential for pathogen defense and inflammatory response (Arpaia et al., 2013; Kim et al., 2014; Bui et al., 2025). Bui et al. (2025) recently summarized the effects on T-lymphocyte activation and differentiation derived from a small number of studies in neonates. Thus, a delicate regulation of pro- and anti-inflammatory mechanisms is necessary to maintain immune homeostasis (Arpaia et al., 2013; Smith et al., 2013).

Mao et al. (2022) investigated the effects of *Bifidobacterium* strains with the ability to ferment 2’-FL on the production of SCFA and pro- and anti-inflammatory cytokines in mice. They found that a mix of five strains in the presence of 2’-FL increased the species richness of the gut microbiota, the contents of acetic acid and in particular of isobutyric acid. Whether these metabolites were produced by the *Bifidobacterium* mix they applied as we have seen for *B. longum* subsp. *infantis* and *B. breve* for 6’-SL containing media or are derived from cross-feeding with other species of the intestinal microbiota, remains open. Through these direct effects on specific SCFA, *Bifidobacterium* species may affect the immune system and thus have an impact on infection outcomes in early life or the development of allergies (Bui et al., 2025). But whether this may explain in part the increased allergy risk associated with caesarian section and concomitant differences in the newborn microbiota with lower abundances of *Bifidobacterium* species (Arboleya et al., 2016) remains an open question.

Samarra et al. (2025) recently reported that *B. bifidum* demonstrated the strongest HMO-degrading ability but had the lowest antibiotic resistance among bifidobacteria. Together with their ability to produce organic acids by metabolizing HMOs such as 2’-FL as well as 6’-SL, even in lower cell numbers as shown in this study, the sensitivity of bifidobacteria to antibiotics emphasizes the need for a more restrained prescription of antibiotics and continued breastfeeding promotion. The importance of colonization, i.e., the order of arrival of *Bifidobacterium* species and the presence of HMOs via breastfeeding as a selective force for promoting the establishment of a beneficial microbiota has been underlined by Laursen and Roager (2023). They showed that when mice were supplemented with HMOs, the strength of priority effects diminished, and *B. longum* subsp. *infantis* dominated regardless of colonization order. The low prevalence reported for many Western populations as they have shown in 25 breastfed Danish infants over the first 6 months of life with 11 out of 25 breastfed infants with highly abundant *B. longum* subsp. *infantis* (Laursen and Roager, 2023).

The present findings suggest a synergistic effect of different carbohydrate substrates, particularly at higher concentrations, on organic acid production when these substrates can be utilized by all *Bifidobacterium* strains. However, when the effects of cross-feeding are taken into account (Katoh et al., 2020), specific strains such as *B. bifidum* enable others to grow and to produce even higher amounts of organic acids together in co-culture by using substrates such as 2’-FL and 6’-SL they are not able to use in single strain cultures.

This may represent an adaptive mechanism that enables bifidobacteria to exploit a broader range of substrates in the gastrointestinal tract of breastfed infants, where the composition of HMOs is highly diverse. In conclusion, our findings demonstrate the growth of infant-associated *Bifidobacterium* strains on HMOs and lactose or monosaccharides does not reliably predict the quantity and profile of fermentation metabolites produced. Using defined single- and multi-strain cultures, we show that cross-feeding interactions allow low-abundance strains, particularly *B. bifidum*, to exert a disproportionate influence on SCFA production.

These results have direct implications for pro- and synbiotic formulation as has also been emphasized by De Bruyn et al. (2024). Inclusion of strains with complementary enzymatic functions may be critical for enabling utilization of specific HMOs such as 6’-SL and for efficient production of organic acids, even when such strains do not dominate numerically.

Together, these observations highlight the importance of considering strain interactions and substrate combinations, rather than growth alone, when designing synbiotics intended to support early gut microbial development.

## Data Availability

The original contributions presented in the study are included in the article/Supplementary material, further inquiries can be directed to the corresponding author.
