# Supplementary material for: Metabolic interactions and growth dynamics of Bifidobacterium strains in response to human milk oligosaccharides and lactose
Source: Front Microbiol. 2026 Sep 1;17:1805620. doi: 10.3389/fmicb.2026.1805620 (PMC13574977; doi:10.3389/fmicb.2026.1805620)
Supplement: Supplementary file 1 [file Table_1.docx]

**Supplement material S1**

**Metabolic interactions and growth dynamics of *Bifidobacterium* strains in response to human milk oligosaccharides and lactose**

**Stefan Ratering ^1^, Sylvia Schnell ^1^, Christian Borsch ^2^, Sinéad T. Morrin ^3^, David R. Hill ^3^, Rachael H. Buck ^4^, Silvia Rudloff ^2, 5*^**

Figure S1.1: Calibration of the optical density (OD) of *B. longum* subsp. *infantis* against the number of copies per mL. As this is a single-copy gene, the copy number of the gene can be taken to be equal to the number of bacteria.

Figure S1.2: Calibration of the optical density (OD) of *B. breve* against the number of copies per mL. As this is a single-copy gene, the copy number of the gene can be taken to be equal to the number of bacteria.

Figure S1.3: Calibration of the optical density (OD) of *B. bifidum* against the number of copies per mL. As this is a single-copy gene, the copy number of the gene can be taken to be equal to the number of bacteria.

Figure S1.4: Spearman correlations for non-normally distributed data or Pearson correlation for normal distributed data of growth (OD) and output of organic acids (OA) in the different cultures.

Figure S1.5: Heatmap (Manhattan, average) of organic acids concentrations after bacterial fermentation in the supernatant of culture media supplemented with HMO (2´-FL, 6´-SL, lactose or glucose) as indicated. Average organic acids concentrations from 3 experiments are given in mM.

Table S1.1: Copy numbers for the inoculation of the mixed culture experiments. Once the OD had been determined, the copy number per mL was calculated using the standard curves (Fig. S1.1–S1.3). These values were then used to calculate and prepare the mixture. After thorough mixing, the cultures for the experiments shown in Figs. 2–3 were inoculated from this mixture. The lowercase ‘l’ and ‘r’ in the figure number refer to the right and left graphs of the qPCR results in Figure 2.

| Figure | Copy numbers ml^-1^  *B. l. subsp. infantis* | Copy numbers ml^-1^  *B. breve* | Copy numbers ml^-1^  *B. bifidum* | Mixing ratio  (ml) |
| --- | --- | --- | --- | --- |
| Fig. 2Ar | 3.07 x 10^8^ | 7.89 x 10^8^ | ---- | 7.8 + 5 |
| Fig. 2Br | 3.07 x 10^8^ | ---- | 3.67 x 10^8^ | 7.2 + 6 |
| Fig. 2Cr | ---- | 8.38 x 10^8^ | 3,78 x 10^8^ | 5 +11.1 |
| Fig. 3 | 2.83 x 10^8^ | 7.18 x 10^8^ | 3.49 x 10^8^ | 20.3 + 8 +16.4 |
| Fig. 2Al | 2.86 x 10^8^ | 6.47 x 10^8^ | ---- | 4 + 8.94 |
| Fig. 2Bl | 3.33 x 10^8^ | ----- | 4.15 x 10^8^ | 7.5 +6 |
| Fig. 2Cl | ---- | 8.38 x 10^8^ | 3,78 x 10^8^ | 5 +11.1 |
